# Serum artemin is not correlated with sensitivity within dogs with naturally occurring osteoarthritis pain

**DOI:** 10.1038/s41598-021-85976-y

**Published:** 2021-03-23

**Authors:** Ankita Gupta, Ludovica Chiavaccini, Laura M. Minnema, King Wa Chiu, David Knazovicky, Jonathan A. Hash, Santosh K. Mishra, B. Duncan X. Lascelles

**Affiliations:** 1 Translational Research in Pain Program, North Carolina State University, Raleigh, NC 27606 USA; 2 Department of Clinical Sciences, College of Veterinary Medicine, North Carolina State University, Raleigh, NC 27606 USA; 3 Department of Molecular Biomedical Sciences, College of Veterinary Medicine, North Carolina State University, Raleigh, NC 27606 USA; 4 Comparative Pain Research and Education Centre, North Carolina State University, Raleigh, NC 27606 USA; 5 Thurston Arthritis Center, University of North Carolina At Chapel Hill, Chapel Hill, NC 27599 USA; 6 Center for Translational Pain Medicine, Duke University, Durham, NC 27710 USA

**Keywords:** Musculoskeletal system, Neuroscience, Sensory processing

## Abstract

Osteoarthritis (OA) pain is associated with peripheral and central sensitization in humans and results in widespread increased sensitivity across the body. Sensitization contributes to the OA-associated pain (OAP) state. We recently identified increased levels of an endogenous neurotrophic factor, artemin (ARTN), in dogs with OAP compared to healthy pain-free controls. Circulating ARTN released from damaged tissues in OA, may play a central role in widespread sensitivity and pain. However, the relationship between ARTN and somatosensory sensitivity remains unknown. The study aimed to assess the relationship between serum ARTN concentrations and measures of sensitivity in dogs with OAP using quantitative sensory testing. We hypothesized that there would be a positive association between circulating ARTN and increased sensitivity to mechanical and thermal stimuli in dogs with OAP. We used linear and logistic regression models to assess the relationship between ARTN, sensitization, and pain within a cohort of 43 dogs with spontaneous OAP. Serum ARTN was not associated with the degree of sensitization within dogs with OAP. Further, across dogs with varying OAP severity, we did not find any association between ARTN, and clinical measures of joint pain and disability. Although a relationship between ARTN and joint pain was not ruled out.

## Introduction

Osteoarthritis (OA) is the most common form of arthritis and is a major cause of pain and disability in nearly 30 million adults in the U.S ^1^. It is characterized as a chronic pain condition and is associated with peripheral (PS) and central sensitization (CS) ^2–4^. Together, PS and CS can be referred to as generalized sensitization (GS), or a generalized algoplastic state. In GS, the nociceptive neurons of the central nervous system are increasingly responsive to normal or subthreshold nerve stimuli, and this contributes to the difficulty in controlling OA-associated pain (OAP) ^5–7^.

Generalized sensitivity in long-standing pain states such as OAP can arise from a wide variety of underlying mechanisms. It can be associated with one or more of the following: sensitization of the peripheral primary afferent neurons ^8^, algoplastic changes within the spinal cord or brain ^8^, or decreased function of the endogenous pain modulation system ^3^. In conditions such as OAP, the GS state is likely established and maintained by peripheral input generated by sensitized and hyperresponsive peripheral afferents. The mechanisms involved in establishing and maintaining functionally upregulated primary afferent neurons are being elucidated in the search for therapeutic targets.

Although a developing area of research, neurotrophins such as nerve growth factor have been implicated in the induction and maintenance of central sensitization. Another neurotrophic factor of interest is artemin (ARTN), a member of the glial cell line-derived neurotrophic factor (GDNF) family. Previous work in rodent models has shown that ARTN can activate bone primary afferents and can induce mechanical ^9,10^ and thermal sensitization ^11–13^. Several lines of evidence indicate ARTN can upregulate TRP channel expression and activity ^14–17^, and these mechanisms appear to be partly responsible for the association between ARTN and increased mechanical, hot, and cold sensitivity. Recent work has shown that anti-ARTN antibodies can both prevent the establishment of complete Freund’s adjuvant (CFA)-induced mechanical sensitivity, and also reverse established CFA-associated mechanical sensitivity, suggesting that ARTN may be involved in both the establishment and maintenance of sensitivity ^10^. Widespread mechanical, hot, and cold thermal hypersensitivity occurs in human patients with OAP ^3,18–22^. Canine spontaneous OA and OAP closely resembles human OA, and client-owned dogs with OA show GS in association with OAP ^23^ as humans do ^3,21^.

We recently found that serum ARTN concentrations are elevated in dogs with naturally occurring OA and OAP, compared to healthy controls without OAP. Higher serum ARTN concentrations were also associated with higher scores on validated clinical metrology instruments (CMIs), which are indicative of pain and disability in pet dogs with OA. We also observed a significant negative relationship between synovial fluid ARTN levels and limb use in dogs with OAP ^24^. Additionally, the receptor for ARTN, GDNF family receptor alpha-3 (GFRα3), was found to be upregulated in the dorsal root ganglia serving painful OA joints in dogs. To date, no studies in any species have investigated the relationship between circulating ARTN and sensitization.

Given that serum ARTN is increased in dogs with OAP, and dogs with OAP have increased sensitivity, we now wanted to explore if there was a possible causal relationship between serum ARTN and sensitivity. The primary aim of this study was to assess the relationship between serum ARTN concentrations and somatosensory sensitivity measured using quantitative sensory testing (QST) within dogs with naturally occurring OAP. We wanted to determine if, within dogs with OAP, serum ARTN concentrations were associated with increased sensitivity. We were not looking for a biomarker, but rather looking to determine if there was a positive relationship between serum ARTN and sensitivity that may suggest a causal relationship. Our secondary objectives were to determine if there was an association between serum ARTN and measures of pain and disability. We hypothesized that there would be a positive relationship between serum ARTN and sensitivity to mechanical and thermal stimuli, and further, we hypothesized that serum ARTN would be significantly associated with clinical measures of pain and disability. Understanding the role of ARTN in OAP may lead to the development of novel therapies for pain.

## Results

A total of 43 dogs with OAP were recruited for this study. The sample size was determined based on data availability from two prospective studies (^23^, unpublished data). No pilot data was available to perform a sample size estimation for this exploratory study. Descriptive statistics for patient demographics, orthopaedic exam findings, CMI scores, QST thresholds and latencies, and serum ARTN concentrations are listed in Table 1 and Supplementary Table S1. Data from subjects whose owners failed to complete the Liverpool Osteoarthritis in Dogs (LOAD) and the Canine Brief Pain Inventory (CBPI) surveys successfully were excluded from the CMI analysis. Thus, the sample size for the LOAD and CBPI survey were 41 and 42 dogs, respectively. Most dogs in this study had concurrent hip and stifle OA. Serum samples were collected at a mean of 0.5 days (+ / − 1.7) after QST was performed: 39 samples were collected at the time of QST; 1 was collected 4 days, 2 at 6 days and 1 at 7 days after QST.

**Table 1:** Patient demographics of recruited dogs with naturally occurring osteoarthritis-associated pain.

	Variable	OA group (mean ± standard deviation; median, (Q1, Q3) or sample size (n, %))
Demographics (n = 43)	Age (years)	8.16 ± 3.15; 8, (6,10)
	Sex	Male intact (n = 2, 4.65%)
		Male neutered (n = 19, 44.19%)
		Female intact (n = 0, 0.00%)
		Female spayed (n = 22, 51.16%)
	Body weight (kgs)	30.65 ± 8.56; 28.40, (25.9–33.8)
	Body condition score	Score 4: (n = 7, 16.28%)
		Score 5: (n = 18, 41.86%)
		Score 6: (n = 12, 27.91%)
		Score 7: (n = 4, 9.30%)
		Score 8: (n = 2, 4.65%)
QST Values (n = 43)	Mechanical threshold (g)	1275.85 ± 430.64; 1294.20, (1011.60, 1584.60)
	Thermal latency (secs)	15.64 ± 5.13; 17.92, (10.68, 20.00)
	Rater or investigator	KWC (n = 35, 81.39%)
		DK (n = 8, 18.60%)
	Feasibility score	Score 0: (n = 40, 93.02%)
		Score 1: (n = 1, 2.33%)
		Score 3: (n = 2, 4.65%)
Orthopaedic Exam Findings (n = 43)	Grouped joint pain score	Mild: (n = 11, 25.58%)
		Moderate: (n = 14, 32.56%)
		High: (n = 11, 25.58%)
		Severe: (n = 7, 16.28%)
	Grouped muscle atrophy score	Mild: (n = 13, 30.23%)
		Moderate: (n = 9, 20.93%)
		High: (n = 12, 27.91%)
		Severe: (n = 9, 20.93%)
	Index side	Left side (n = 18, 41.86%)
		Right side (n = 25, 58.14%)
CMI Scores (n = 41 or 42)	LOAD score (n = 41)	20.80 ± 7.81; 21, (14.50, 26.50)
	CBPI PSS (n = 42)	3.28 ± 1.69; 3.37, (1.75, 4.50)
	CBPI PIS (n = 42)	4.15 ± 2.33; 4.75, (2.00, 6.00)
Serum analysis (n = 43)	ARTN Concentration (ng/mL)	2.26 ± 1.92; 1.53, (0.91, 3.66)

Full descriptive statistics (mean, standard deviation, median, and (Q1, Q3), or n and %) for patient demographics (age, sex, body weight, and body condition score), QST (mechanical threshold, thermal latency, rater, and feasibility score), orthopaedic exam findings (grouped joint pain and muscle atrophy scores and index side), CMI values (LOAD score, CBPI PSS and CBPI PIS), and serum ARTN concentrations in dogs with naturally occurring OA . Data is displayed as (mean ± SD; median, (Q1, Q3)) for continuous variables, or the sample size (n, %) is listed for ordinal and nominal variables. Breed descriptive statistics are listed in Supplementary Table S1. OA, Osteoarthritis; QST, Quantitative Sensory Testing; CMI, Clinical Metrology Instruments; LOAD, Liverpool Osteoarthritis in Dogs; CBPI PSS, Canine Brief Pain Inventory Pain Severity Score; CBPI PIS, Canine Brief Pain Inventory Pain Interference Score; ARTN, artemin.

### Relationship between somatosensory sensitivity and ARTN

**Figure 1:**
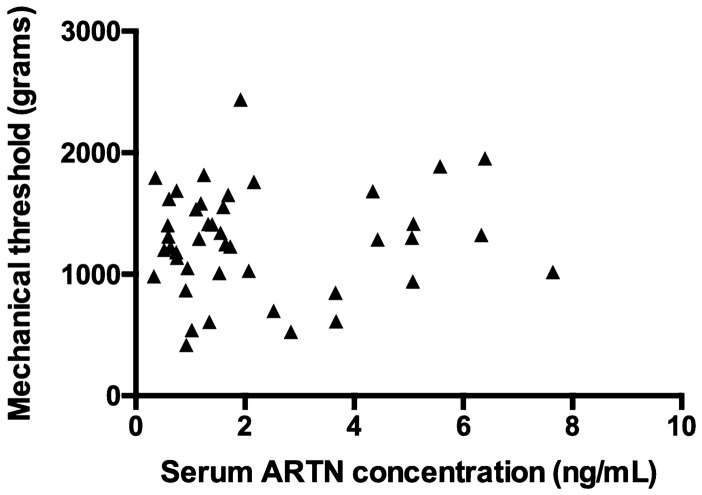
Scatterplot of serum ARTN concentrations (ng/mL) versus mechanical threshold (g) in dogs with naturally occurring osteoarthritis-associated pain (r = 0.05, *P* = 0.73). ARTN, artemin.

Body weight and the rater performing the exam showed a weak association (*P* ≤ 0.20) with mechanical threshold and were included in the multivariable model. Age, sex, body condition score (BCS), and feasibility score were not associated with the mechanical threshold outcome. ARTN serum concentration, although not associated with the outcome (*P* = 0.71) was forced into the model as the variable of interest (Table 2). The final model included body weight and serum ARTN concentrations and was overall significant (*P* < 0.01) (Table 3). When considering all the variables in the model, the mechanical threshold increased approximately 24 grams (g) for every kg increase in body weight (coeff. 24.38 g, SE 6.95, 95% CI: 10.32–38.44, *P* < 0.01). However, serum ARTN concentrations were not associated with mechanical threshold (coeff. 19.88 g, SE 31.65, 95% CI: − 44.14–83.91, *P* = 0.53). A Spearman correlation analysis showed that there was no significant relationship between mechanical threshold and serum ARTN concentrations (Fig. 1) and neither between body weight and serum ARTN concentrations (Supplementary Figure S1).

**Table 2:** Standard (ordinal) least squares model of the association between univariate variables and mechanical threshold (g) in dogs with naturally occurring osteoarthritis-associated pain (* indicates *P* < 0.05).

Variable	Coefficient	Standard error	95% Confidence interval	*P* value
Serum ARTN concentrations	13.19	34.89	− 57.26–83.65	0.71
Body weight	24.34	6.87	10.46–38.22	< 0.01*
Rater				0.15
KWC	Reference	–	–	–
DK	− 121.58	83.27	− 289.74–46.58	0.15
Age	− 19.68	21.15	− 62.39–23.04	0.36
Sex				0.31
Male Neutered	Reference	–	–	–
Male intact	304.48	206.86	− 113.60–722.56	0.15
Female Spayed	− 116.02	122.39	− 363.38–131.34	0.35
Body condition score				0.49
5–4	− 161.31	192.96	− 551.93–229.31	0.41
6–5	− 91.62	161.44	− 418.44–235.19	0.57
7–6	− 55.73	250.10	− 562.03–450.57	0.82
8–7	527.78	375.15	− 231.67–1287.23	0.17
Feasibility score				0.53
1–0	408.53	439.81	− 480.36–1297.41	0.36
3–1	− 600.28	532.04	− 1675.58–475.02	0.27

As the variable increases, the mechanical threshold value increases (positive) or decreases (negative) by the coefficient value. ARTN, artemin.

**Table 3:** Standard (ordinal) least squares model of the association between multivariate variables and mechanical threshold (g) in dogs with naturally occurring osteoarthritis-associated pain (* indicates *P* < 0.05).

Variable	Coefficient	Standard error	95% Confidence interval	*P* value
Model				< 0.01*
Serum ARTN concentrations	28.86	30.93	− 33.66–91.38	0.36
Body weight	25.25	6.95	11.420– 39.30	< 0.01*

As the variable increases, the mechanical threshold value increases by the coefficient value. ARTN, artemin.

**Figure 2:**
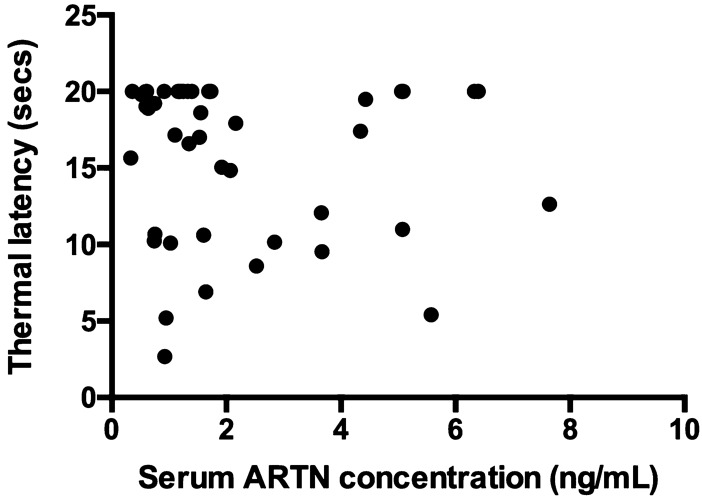
Scatterplot of serum ARTN concentrations (ng/mL) versus thermal latency (secs) in dogs with naturally occurring osteoarthritis-associated pain (r = − 0.09, *P* = 0.57). ARTN, artemin.

Age, sex, body weight, BCS, rater, and feasibility score were not associated with thermal latency. Serum ARTN concentration was not associated with thermal latency (coeff. − 0.20 secs, SE 0.41, 95% CI: − 1.04–0.63, *P* = 0.63, Table 4). A Spearman correlation analysis showed that there was no significant relationship between thermal latency and serum ARTN concentrations (Fig. 2).

**Table 4:** Standard (ordinal) least squares model of the association between univariate variables and thermal latency (secs) in dogs with naturally occurring osteoarthritis-associated pain.

Variable	Coefficient	Standard error	95% Confidence interval	*P* value
Serum ARTN concentrations	− 0.11	1.02	− 2.17–1.95	0.92
Body weight	0.22	0.23	− 0.23–0.68	0.33
Rater				0.20
KWC	Reference	–	–	–
DK	− 3.15	2.44	− 8.08–1.78	0.20
Age	0.39	0.62	− 0.85–1.65	0.53
Sex				0.41
Male Neutered	Reference	–	–	–
Male intact	7.28	6.07	− 5.00–19.57	0.24
Female Spayed	− 4.81	3.59	− 12.07–2.46	0.19
Body condition score				0.53
5–4	− 6.13	5.64	− 17.56–5.29	0.28
6–5	3.44	4.72	− 6.12–13.00	0.47
7–6	− 10.17	7.32	− 24.98–4.64	0.17
8–7	5.5	10.97	− 16.72–27.72	0.62
Feasibility score				0.59
1–0	− 1.48	12.86	− 27.46–24.51	0.91
3–1	− 8	15.55	− 39.43–23.43	0.61

As the variable increases, the thermal latency value increases or decreases by the coefficient value. ARTN, artemin.

A Spearman correlation analysis showed that there is a significant moderate correlation between mechanical threshold (g) and thermal latency (secs) (Supplementary Figure S2, r = 51, *P* < 0.001).

### Relationship between orthopaedic exam findings and ARTN

**Figure 3:**
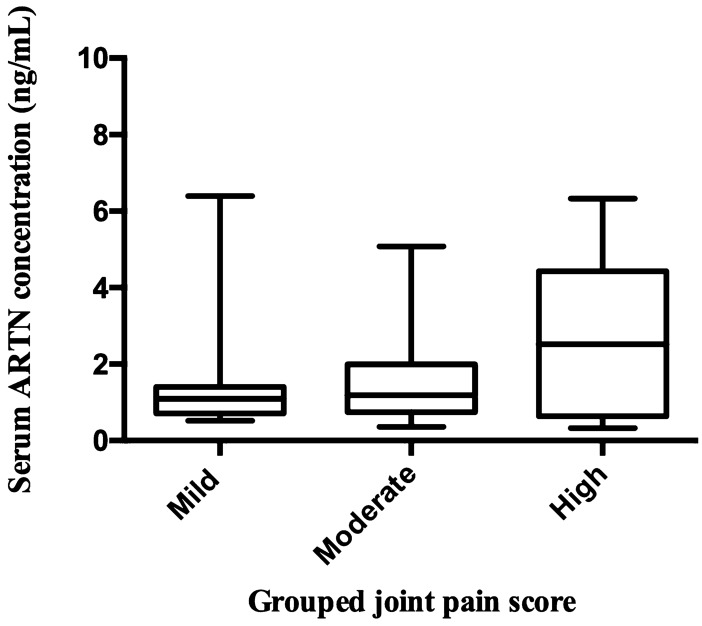
Box and whisker plot of grouped joint pain scores (mild (total score 2–4), moderate (total score 5–6) and high (total score 7–9)) versus serum ARTN concentrations (ng/mL) in dogs with naturally occurring osteoarthritis-associated pain. ARTN, artemin.

The scores of the four limbs were combined to create total joint pain (range 2–22 points) and muscle atrophy scores (range 2–21 points) and then classified as mild-severe groups. There was no association between the grouped joint pain score (chi-square 0.45, R^2^ < 0.01, *P* = 0.50) or grouped muscle atrophy score (chi-square 0.89, R^2^ < 0.01, *P* = 0.34) and serum ARTN concentrations (Table 5). However, visual assessment of the data suggested a possible relationship between serum ARTN and joint pain for the mild-moderate-high categories. After removing the severe joint pain category and outliers that were more than two standard deviations from the mean, we re-evaluated the relationship between grouped joint pain score and serum ARTN concentrations. No significant relationship was detected at *P* value < 0.05 but the association was significant at an alpha level of 0.1 (chi-square 3.44, R^2^ = 0.05, *P* = 0.06, Table 5, Fig. 3).

**Table 5:** Ordinal logistic analysis model of the association between grouped joint pain score, grouped muscle atrophy score, and serum ARTN concentrations in dogs with naturally occurring osteoarthritis-associated pain.

Orthopaedic exam scores	Chi-square	R^2^	*P* value
Grouped joint pain score	0.45	< 0.01	0.50
Grouped muscle atrophy score	0.89	< 0.01	0.34
Grouped joint pain score (without severe pain category and outliers)	3.44	0.05	0.06

We evaluated the relationship between grouped joint pain score and serum ARTN concentrations after dropping the severe joint pain category and outliers. ARTN, artemin.

### Relationship between CMI scores and ARTN

There was no association between the LOAD score (coeff. 0.77, SE 0.63, 95% CI: − 0.51–2.05, *P* = 0.23), CBPI Pain Severity Score (PSS) (coeff. 0.12, SE 1.00, 95% CI: − 1.90–2.15, *P* = 0.90) or CBPI Pain Interference Score (PIS) (coeff. 0.28, SE 1.00, 95% CI: − 1.74–2.31, *P* = 0.78) and serum ARTN concentration (Table 6).

**Table 6:** Standard (ordinal) least squares model of the association between CMI scores (LOAD, CBPI PSS and CBPI PIS) and serum ARTN concentrations in dogs with naturally occurring osteoarthritis-associated pain.

Variable	Coefficient	Standard error	95% Confidence interval	*P* value
LOAD score	0.77	0.63	− 0.51–2.05	0.23
CBPI PSS	0.12	1.00	− 1.90–2.15	0.90
CBPI PIS	0.28	1.00	− 1.74–2.31	0.78

CBPI PSS and PIS were rank transformed using the column rank function. As the variable increases, the serum ARTN concentration increases by the coefficient value. CMI, Clinical Metrology Instruments; LOAD, Liverpool Osteoarthritis in Dogs; CBPI PSS, Canine Brief Pain Inventory Pain Severity Score; CBPI PIS, Canine Brief Pain Inventory Pain Interference Score; ARTN, artemin.

## Discussion

The main objective of this study was to evaluate the association between serum ARTN concentrations and measures of somatosensory sensitivity using QST in dogs with spontaneously occurring OAP. The results of this study suggest that, *within* a population of dogs with OAP, there is no significant relationship between ARTN and mechanical threshold or thermal latency, measured using QST. That is, within dogs with naturally occurring OAP, measurable sensitivity does not increase as serum ARTN increases. This result does not negate a role of ARTN in establishing or maintaining OA-pain associated sensitivity, but rather concludes that higher serum ARTN concentrations do not appear to lead to greater sensitivity. To our knowledge, no studies have investigated the relationship between serum ARTN concentrations, and mechanical or thermal hypersensitivity associated with OAP.

We used a naturally occurring canine model of OAP—owned, pet dogs with OAP. Dogs develop OA and OAP naturally, and outcome measures have been well established in this species ^25,26^. The canine naturally occurring model of OA and OAP closely resembles human OA and OAP biology, pathology ^27^, and genetics ^28^. Further, GS occurs in association with OAP in dogs ^23^ as it does in humans ^3,21^. Recent reviews have highlighted that the naturally occurring veterinary model of OAP is a highly relevant translational model for human medicine and useful in the process of developing and validating targets for pain therapeutics ^29,30^.

ARTN, has recently been identified as playing a role in acute inflammatory bone pain ^9^ and a potential role in naturally occurring OAP ^24^. We have previously reported that serum ARTN concentrations are elevated in dogs with OAP compared to controls (*P* ≤ 0.05). We also identified associations between serum ARTN and overall measures of pain and disability using the CBPI measure (*R*^2^ = 0.16; *P* = 0.014), and synovial fluid ARTN and joint pain, indicated by limb use (*R*^2^ = 0.62; *P* = 0.02) ^24^. ARTN binds to its receptor GFRα3 on primary afferent (nociceptive) neurons and contributes to the formation of the GDNF receptor complex with RET receptor tyrosine kinase ^31,32^ initiating a downstream signalling path, as attested by activation of the mitogen-activated protein kinase pathway ^31^. GFRα3 is primarily expressed in peptidergic neuronal populations and is co-expressed with transient receptor potential (TRP) ion channel proteins such as TRPV1, TRPA1, and TRPM8 in the dorsal root ganglia (DRG) and trigeminal ganglia ^16,32,33^. These TRP channels are involved in encoding hot, mechanical, and cold sensations. Animal models of OA have been utilized to demonstrate the upregulation of TRP channel activity in sensitized states ^34–37^. Repeated in vivo ARTN injections into the peripheral hind paw of the mouse can change TRPV1 and TRPA1 gene expression at the level of the DRG ^14^, and other work has shown that a one-time hind paw ARTN injection in adult male mice can lead to hyperalgesia and sensitize thermal nociceptors ^11^. Blockade of ARTN with a monoclonal antibody can partially reverse mechanical hypersensitivity in the CFA  mouse model of inflammatory pain ^38^. ARTN, like other neurotrophins, plays an upstream role in defining the sensitivity of the primary afferent fibre. Together, these findings support the idea that ARTN may play a role in widespread sensitization. However, no studies had hitherto evaluated the association between ARTN, and mechanical and thermal sensitization associated with OAP until now.

QST is commonly used in human medicine to quantify alterations in nociceptive processing semi-objectively ^39,40^. It can be used to measure changes in pain sensitivity via the application of a mechanical or thermal test stimulus to a peripheral site and recording the subject’s response. In the naturally occurring canine model of OAP, QST was used to test for widespread somatosensory sensitivity ^23^ as found in humans with OAP ^41–43^. Compared to healthy control dogs without OAP, dogs with OAP were found to have widespread sensitivity to mechanical, hot, and cold stimuli. Although studies have not evaluated the relationship between sensitivity to different modalities, previous work has suggested generally increased sensitivity to all modalities ^23^ and indeed, in the current cohort, there was a positive correlation between mechanical and hot thermal sensitivity. Due to the known role of ARTN in modulating the sensitivity of primary afferent fibres, primarily via TRP channels, we wanted to assess the relationship between ARTN and QST profiles. Our results indicated that there is no association between circulating serum ARTN and QST profiles across a cohort of dogs with OAP. However, it must be remembered that if ARTN is involved in OAP, then locally produced ARTN in the affected joint/bone tissues is likely to have a more direct relationship to neuronal sensitivity and subsequent PS and CS. Additionally, the state is dependent upon many factors, including the peripheral neuronal changes and input, spinal cord processes and descending controls, and it is probably rather naïve to think the concentrations of a single ligand would be related to sensitivity.

Our statistical models indicated that the dominant factor affecting the mechanical threshold of dogs with OAP is body weight. Studies by other prominent research groups have also noted a positive association between the mechanical threshold and body weight of healthy control ^44–47^, OA ^48^, and when comparing both control and OA dogs ^48^ using a blunt-tipped handheld pressure algometer ^44,45,47,48^ or an electronic von Frey ^45,46^. Interestingly, studies in other animal species, such as healthy 1–2 week old Landrace x Yorkshire piglets ^49^ and domestic cats ^50^, have also found a similar positive relationship between body weight and the mechanical threshold at axial and appendicular sites using the blunt-tipped handheld pressure algometer ^49,50^ or an electronic von Frey ^50^. In the present study, we used a multivariable regression analysis to control for the possible confounding effect of body weight on the mechanical threshold. We considered body weight as a continuous variable based on our large study sample size and as evidenced by other studies ^44,46–50^. Future studies should investigate the effect of categorized body weight and ARTN on the mechanical threshold. By scaling mechanical threshold values to body weight and then comparing them with ARTN serum concentrations may produce a standardized range of values for dogs with OAP for different small, medium, and large body weight groups. Despite our large sample size, we did not have a sufficient number of animals for the small and large body weight groups. Further, there was no significant relationship between body weight and serum ARTN concentrations.

Validated CMIs such as the LOAD and CBPI scores are indicative of owner evaluations of pain and mobility impairment in pet dogs. Previous work by our research group found a weak positive correlation between serum ARTN concentrations and higher CMI scores across healthy dogs and dogs with OAP ^24^, suggesting increased serum ARTN concentrations are weakly associated with greater disability in dogs with OAP. Interestingly, in the present study, we did not identify any relationship between serum ARTN concentrations and CMI scores. This can be explained by the fact that we only investigated CMI scores within the diseased OAP canine study sample. However, when both healthy control and dogs with OAP are examined together, then a relationship is evident between serum ARTN concentrations and CMI scores simply explained by the fact that serum ARTN concentrations and CMI scores are lower in healthy non-OAP dogs, and they are both raised in OAP dogs.

Overall, there was no significant association between serum ARTN and grouped joint pain scores. Still, our data does suggest a relationship between the two over the range of mild, moderate and high joint pain (as we defined it). Our data suggests a relationship, and is compatible with previous findings, although further work is needed to evaluate this fully.

A limitation of the present study is that no healthy controls were included, limiting our ability to draw firm conclusions about the role of ARTN in sensitivity associated with OAP. Additionally, we only evaluated one point in time. Most serum samples for ARTN assessment (n = 39) were collected on the same day QST testing was performed. However, not all ARTN and QST assessments (n = 4) were precisely paired as there was up to a 7-day gap between serum collection and QST assessment. However, previous work (unpublished, associated with ^24^, indicates that serum ARTN concentrations do not change over the period of 1 week). Together, our data and published information indicate ARTN may play a role in the establishment and/or maintenance of OAP, but within OAP, serum ARTN does not appear to be the driver of sensitivity. Future work should evaluate ARTN in GS states when both control and OAP subjects are evaluated together and look at changes over time and different stages of the disease. Another limitation is that QST data was collected by two different raters, KWC and DK. To account for inter-rater variability, both raters were trained by BDXL and had at least five years of advanced veterinary clinical experience. Further, the univariate analysis showed no significant effect between rater and mechanical threshold or thermal latency.

## Methods

Data and samples were used from two prospective clinical studies (^23^, and unpublished data), both approved by the Institutional Animal Care and Use Committee (ethical oversight) at North Carolina State University (protocol numbers: 11-073-O and 16-186-O). All animal work was conducted according to the guidelines outlined in the Animal Welfare Act of 1966 and the Health Research Extension Act of 1985. These acts regulate the basic standards for well-being, care, and treatment of animals for biomedical research. This study was carried out and reported in compliance with the ARRIVE and CONSORT guidelines.

### Subject recruitment

**Figure 4:**
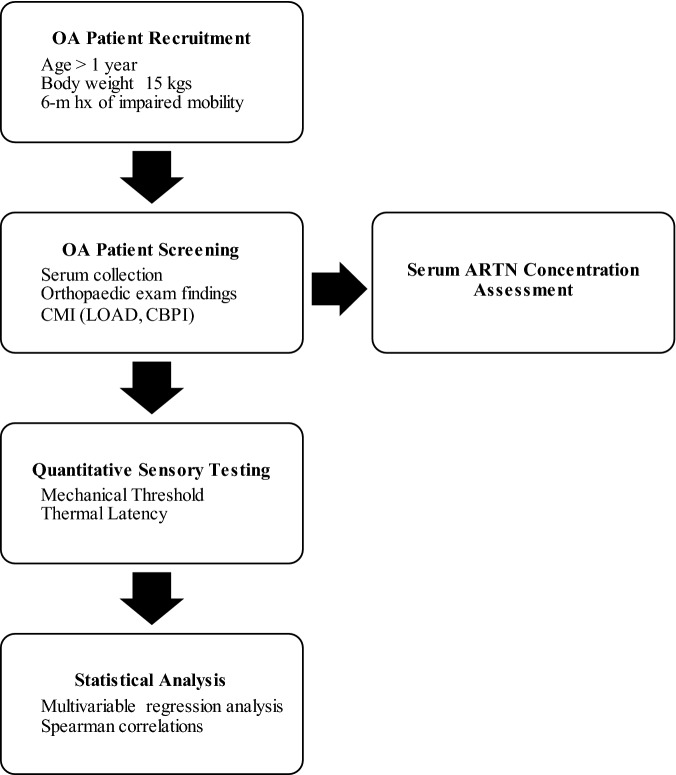
Flowchart describing the study schedule. OA, osteoarthritis; CMI, Clinical Metrology Instruments; LOAD, Liverpool Osteoarthritis in Dogs; CBPI, Canine Brief Pain Inventory; ARTN, artemin.

Client owned dogs affected by OA and OAP were recruited by the staff of the Translational Research in Pain Program with the assistance of the Clinical Studies Core at the North Carolina State University using email, flyers, and through local newspaper and social media advertisements. Both written and verbal consent was obtained from all owners before any procedures were performed. The study flow is displayed (Fig. 4 and Supplementary Figure S3) and described in detail in the following sections.

### Study inclusion criteria

*We only included subjects* older than one year of age and weighing at least 15 kilograms (kgs); with at least a 6-month history of impaired mobility, radiographic evidence of OA in the pelvic and thoracic limb(s) and showing pain on manipulation of at least one joint with radiographic evidence of OA. Subjects were required not to be receiving any analgesics (e.g., NSAIDs), or have had a minimum 3-week washout of known or putative analgesic medications. Nutritional supplements were permitted if the dog had been administered these for six weeks before the study. Only animals who met all the inclusion criteria were enrolled in the study. Dogs were excluded if there was evidence of clinically detectable neurologic disease or any other systemic disease that could be associated with pain.

### Screening

The screening appointment took place at the Health and Wellness Clinic at North Carolina State University, College of Veterinary Medicine. Subject demographics (age, sex, breed, body weight, and BCS were collected at presentation. Complete blood count, serum biochemical analysis, and a freely voided urinalysis were performed simultaneously for each subject to rule out the presence of any underlying systemic diseases. Serum samples were collected and frozen at -80 degrees Celsius within two hours after collection and stored for up to ~ 24 months before the ARTN concentration assessment. At admission, a comprehensive hospital exam was performed by two lead investigators (KWC and DK, both trained by BDXL) to confirm the presence of OAP. Full physical, orthopaedic (details listed in Supplementary Table S2), and neurologic examinations were performed by the same two investigators. Joint pain and muscle atrophy scores were determined, as described previously ^23^, and expressed as total scores.

Additionally, owners were asked to complete CMIs. The CMIs used were the LOAD and CBPI. Both are validated, owner-completed subjective measures of the impact of OAP on the dog’s ability to perform daily activities, and the CBPI asks specifically about the presence of pain. The LOAD is a 13-item instrument with all items reported on a 5-point Likert-type scale. Each item is scored between 0 and 4, and the mobility item scores are added together to give an overall LOAD score (0–52) ^51,52^. The CBPI is a 2-part instrument: the PSS is the arithmetic mean of 4 items scored on an 11-point (0–10) numerical scale, and the PIS is the mean of 6 items similarly scored ^53,54^. The same owner completed both questionnaires.

### Quantitative sensory testing

Mechanical threshold and hot thermal latency QST were performed by KWC or DK (both trained by BDXL) on all subjects on the same day (n = 39) or up to 7 days (n = 4) after the screening appointment (the time when serum was collected). QST testing was performed in the gait laboratory, a quiet room (15 × 4 m) dedicated to the collection of limb use (gait) and QST data. Subjects were first acclimatized to the room for at least 5 min. Fresh water was available ad libitum and treats were occasionally provided. After the acclimatization period, subjects were minimally restrained and were placed in lateral recumbency. The test site was the metatarsus of the index joint limb (the limb where the joint with the highest pain score was located). The two devices were used in random order. QST feasibility scores were calculated for each trial. These scores were indicative of the ease with which QST data could be collected (0–5 scale with 0 = no problem in collecting the data—5 = data was impossible to obtain).

- *Mechanical Threshold*
  A blunt-tipped pressure algometer (SMALGO algometer, Bioseb, Vitrolles, France) with a flat, 3-mm diameter tip attached to a recording unit was used to calculate mechanical pressure thresholds. The device was applied directly, and perpendicular to the subject's metatarsus, placed between metatarsal 2 and 3, or 3 and 4, level at the midpoint of the metatarsus. The force was steadily increased to a maximum of 2500 g. Once a behavioural response was elicited (head turn, limb withdrawal, or vocalization), the test was terminated, and the reading in grams recorded. If no reaction was obtained, a force of 2500 g (maximum) was recorded. The test was repeated five times, with an intertrial interval of 60 secs. The mean threshold in g was calculated and used for data analysis.
- *Thermal Latency*

A thermal probe (NTE-2A; Physitemp Instruments, Clifton, NJ) with a flat, 13-mm diameter tip connected to a digital temperature control unit and recirculating pump and water reservoir was used to provide a hot thermal stimulus. The device delivered a fixed stimulus (49 °C). The probe was applied directly, and perpendicular, to the subject's metatarsus. Once a behavioural response was elicited (head turn, limb withdrawal, or vocalization), the test was stopped, and the latency to response (secs) was recorded. A test cut-off time of 20 s was used to prevent tissue damage. This test was repeated five times with an intertrial interval of 60 secs. The average thermal latency in secs was calculated and used for data analysis.

### Serum artemin concentrations

Serum samples were analysed using a quantitative competitive enzyme-linked immunosorbent assay (ELISA). The Canine ARTN ELISA kit was acquired from ABclonal (ECA0039 (96 tests); ABclonal Inc, Woburn, MA, USA, ^55^). This ELISA kit was validated in-house using mouse Immunoglobulin G (IgG) isotype negative control and mouse ARTN antibodies (PA5-47,063; ThermoFisher Scientific, Waltham, MA, USA). The intensity of the reaction was measured at 450 nm using a Synergy 2 Multi-Mode microplate reader (BioTek Instruments Inc, Winooski, VT, USA). Serum ARTN concentrations (ng/mL) were determined by comparing the optical density of the samples to the optical density of the standard curve (standards were included with the ARTN ELISA kit).

### Statistical analysis

We used JMP Pro 14.1 for Mac (2018 SAS Institute Inc., Raleigh, NC) and GraphPad Prism 6.0c for Mac (GraphPad Software, La Jolla California, USA) for data manipulation, descriptive statistics, regression analysis and for generating figures. We first described patient demographics, QST, orthopaedic exam findings, and CMI. Continuous data were checked for normality using the Shapiro–Wilk normality test, and graphically with quantile–quantile plot and histogram population distribution; data were reported as mean ± SD or median (Q1, Q3) if normally or not normally distributed respectively. Nominal and ordinal data were reported as proportion (%). Thermal latency, CBPI PSS and CBPI PIS did not satisfy the assumption of normality of the distribution and they were rank transformed prior to further analysis. Total joint pain scores were grouped as mild (total score 2–4), moderate (total score 5–6), high (total score 7–9) and severe (total score ≥ 10) based on the data distribution. Similarly, total muscle atrophy scores were classified as mild (total score 2–4), moderate (total score 5–6), high (total score 7–10) and severe (total score ≥ 11). Both the joint pain and muscle atrophy categorization were consistent with the clinical interpretations of mild, moderate, high and severe. The association between ARTN serum concentration and mechanical and thermal latency thresholds, grouped joint pain and muscle atrophy scores, and CMI was studied using multivariable regression analysis. We first performed a univariable analysis for each independent variable that could be associated with the outcome. Factors that showed a weak association with the outcome (*P* ≤ 0.20) were used to build multivariable models that were manually constructed. The significance of categorical variables and meaningful two-way interactions were tested using a likelihood-ratio test. ARTN serum concentration was forced to remain into all models as the primary factor of interest. A scattered plot was used to check for linearity of association between continuous variables and the outcome. The assumptions of homoscedasticity and normal distribution of residuals for mechanical threshold and thermal latency were checked. The assumptions of proportional odds were checked for orthopaedic exam findings and CMI scores. Models were checked for multicollinearity. Additionally, a Spearman correlation analysis was used to assess the relationship between somatosensory sensitivity and serum ARTN concentrations. Significance was set at *P* ≤ 0.05 throughout.

## Supplementary Information

Supplementary Information

## Data Availability

The datasets generated during and/or analysed during the current study are available from the corresponding author on reasonable request.

## References

[1] Cisternas MG (2016). Alternative methods for defining osteoarthritis and the impact on estimating prevalence in a US population-based survey. Arthritis Care Res. (Hoboken).

[2] Bajaj P, Bajaj P, Graven-Nielsen T, Arendt-Nielsen L (2001). Osteoarthritis and its association with muscle hyperalgesia: an experimental controlled study. Pain.

[3] Arendt-Nielsen L (2010). Sensitization in patients with painful knee osteoarthritis. Pain.

[4] Arendt-Nielsen L, Skou ST, Nielsen TA, Petersen KK (2015). Altered central sensitization and pain modulation in the cns in chronic joint pain. Curr. Osteoporos. Rep..

[5] Woolf CJ, Walters ET (1991). Common patterns of plasticity contributing to nociceptive sensitization in mammals and Aplysia. Trends Neurosci..

[6] Woolf CJ, Salter MW (2000). Neuronal plasticity: increasing the gain in pain. Science.

[7] Ji RR, Kohno T, Moore KA, Woolf CJ (2003). Central sensitization and LTP: do pain and memory share similar mechanisms?. Trends Neurosci..

[8] Latremoliere A, Woolf CJ (2009). Central sensitization: a generator of pain hypersensitivity by central neural plasticity. J. Pain.

[9] Nencini S, Ringuet M, Kim DH, Greenhill C, Ivanusic JJ (2018). GDNF, neurturin, and artemin activate and sensitize bone afferent neurons and contribute to inflammatory bone pain. J. Neurosci..

[10] Nencini S, Thai J, Ivanusic JJ (2019). Sequestration of artemin reduces inflammation-induced activation and sensitization of bone marrow nociceptors in a rodent model of carrageenan-induced inflammatory bone pain. Eur. J. Pain.

[11] Malin SA (2006). Glial cell line-derived neurotrophic factor family members sensitize nociceptors in vitro and produce thermal hyperalgesia in vivo. J. Neurosci..

[12] Lippoldt EK, Elmes RR, McCoy DD, Knowlton WM, McKemy DD (2013). Artemin, a glial cell line-derived neurotrophic factor family member, induces TRPM8-dependent cold pain. J. Neurosci..

[13] Lippoldt EK, Ongun S, Kusaka GK, McKemy DD (2016). Inflammatory and neuropathic cold allodynia are selectively mediated by the neurotrophic factor receptor GFRalpha3. Proc. Natl. Acad. Sci. U S A.

[14] Ikeda-Miyagawa Y (2015). Peripherally increased artemin is a key regulator of TRPA1/V1 expression in primary afferent neurons. Mol. Pain.

[15] Elitt CM, Malin SA, Koerber HR, Davis BM, Albers KM (2008). Overexpression of artemin in the tongue increases expression of TRPV1 and TRPA1 in trigeminal afferents and causes oral sensitivity to capsaicin and mustard oil. Brain Res..

[16] Elitt CM (2006). Artemin overexpression in skin enhances expression of TRPV1 and TRPA1 in cutaneous sensory neurons and leads to behavioral sensitivity to heat and cold. J. Neurosci..

[17] DeBerry JJ, Saloman JL, Dragoo BK, Albers KM, Davis BM (2015). Artemin immunotherapy is effective in preventing and reversing cystitis-induced bladder hyperalgesia via TRPA1 regulation. J. Pain.

[18] Graven-Nielsen T, Wodehouse T, Langford RM, Arendt-Nielsen L, Kidd BL (2012). Normalization of widespread hyperesthesia and facilitated spatial summation of deep-tissue pain in knee osteoarthritis patients after knee replacement. Arthritis Rheum..

[19] Neogi T (2015). Sensitivity and sensitisation in relation to pain severity in knee osteoarthritis: trait or state?. Ann. Rheum. Dis..

[20] Finan PH (2013). Discordance between pain and radiographic severity in knee osteoarthritis: findings from quantitative sensory testing of central sensitization. Arthritis Rheum..

[21] Lee YC (2011). Pain sensitivity and pain reactivity in osteoarthritis. Arthritis Care Res. (Hoboken).

[22] Fingleton C, Smart K, Moloney N, Fullen BM, Doody C (2015). Pain sensitization in people with knee osteoarthritis: a systematic review and meta-analysis. Osteoarthritis Cartil..

[23] Knazovicky D (2016). Widespread somatosensory sensitivity in naturally occurring canine model of osteoarthritis. Pain.

[24] Minnema L (2020). Correlation of artemin and GFRalpha3 with osteoarthritis pain: early evidence from naturally occurring osteoarthritis-associated chronic pain in dogs. Front. Neurosci..

[25] Lascelles BDX (2019). Measurement of chronic pain in companion animals: discussions from the pain in animals workshop (PAW) 2017. Vet. J..

[26] Lascelles BDX (2019). Measurement of chronic pain in companion animals: priorities for future research and development based on discussions from the pain in animals workshop (PAW) 2017. Vet. J..

[27] McCoy AM (2015). Animal models of osteoarthritis: comparisons and key considerations. Vet. Pathol..

[28] Maccoux LJ, Clements DN, Salway F, Day PJ (2007). Identification of new reference genes for the normalisation of canine osteoarthritic joint tissue transcripts from microarray data. BMC Mol. Biol..

[29] Klinck MP (2017). Translational pain assessment: could natural animal models be the missing link?. Pain.

[30] Lascelles BDX, Brown DC, Maixner W, Mogil JS (2018). Spontaneous painful disease in companion animals can facilitate the development of chronic pain therapies for humans. Osteoarthritis Cartil..

[31] Baloh RH (1998). Artemin, a novel member of the GDNF ligand family, supports peripheral and central neurons and signals through the GFRalpha3-RET receptor complex. Neuron.

[32] Orozco OE, Walus L, Sah DW, Pepinsky RB, Sanicola M (2001). GFRalpha3 is expressed predominantly in nociceptive sensory neurons. Eur. J. Neurosci..

[33] Goswami SC (2014). Molecular signatures of mouse TRPV1-lineage neurons revealed by RNA-Seq transcriptome analysis. J. Pain.

[34] Barton NJ (2006). Attenuation of experimental arthritis in TRPV1R knockout mice. Exp. Mol. Pathol..

[35] Fernandes ES (2016). Environmental cold exposure increases blood flow and affects pain sensitivity in the knee joints of CFA-induced arthritic mice in a TRPA1-dependent manner. Arthritis Res. Ther..

[36] Fernandes ES (2011). A distinct role for transient receptor potential ankyrin 1, in addition to transient receptor potential vanilloid 1, in tumor necrosis factor alpha-induced inflammatory hyperalgesia and Freund's complete adjuvant-induced monarthritis. Arthritis Rheum..

[37] Galindo T, Reyna J, Weyer A (2018). Evidence for transient receptor potential (TRP) channel contribution to arthritis pain and pathogenesis. Pharm. Basel.

[38] Thornton P (2013). Artemin-GFRalpha3 interactions partially contribute to acute inflammatory hypersensitivity. Neurosci. Lett..

[39] Arendt-Nielsen L, Yarnitsky D (2009). Experimental and clinical applications of quantitative sensory testing applied to skin, muscles and viscera. J. Pain.

[40] Arendt-Nielsen L, Curatolo M (2013). Mechanistic, translational, quantitative pain assessment tools in profiling of pain patients and for development of new analgesic compounds. Scand. J. Pain.

[41] Arendt-Nielsen L (2015). Central sensitization in humans: assessment and pharmacology. Handb. Exp. Pharmacol..

[42] Rolke R (2006). Quantitative sensory testing in the German research network on neuropathic pain (DFNS): standardized protocol and reference values. Pain.

[43] Suokas AK (2012). Quantitative sensory testing in painful osteoarthritis: a systematic review and meta-analysis. Osteoarthritis Cartil..

[44] Harris LK, Murrell JC, van Klink EG, Whay HR (2015). Influence of experimental protocol on response rate and repeatability of mechanical threshold testing in dogs. Vet. J..

[45] Sanchis-Mora S (2017). Development and initial validation of a sensory threshold examination protocol (STEP) for phenotyping canine pain syndromes. Vet. Anaesth. Analg..

[46] Moore SA, Hettlich BF, Waln A (2013). The use of an electronic von Frey device for evaluation of sensory threshold in neurologically normal dogs and those with acute spinal cord injury. Vet. J..

[47] Briley JD, Williams MD, Freire M, Griffith EH, Lascelles BD (2014). Feasibility and repeatability of cold and mechanical quantitative sensory testing in normal dogs. Vet. J..

[48] Harris LK, Whay HR, Murrell JC (2018). An investigation of mechanical nociceptive thresholds in dogs with hind limb joint pain compared to healthy control dogs. Vet. J..

[49] Janczak AM (2012). Factors affecting mechanical (nociceptive) thresholds in piglets. Vet. Anaesth. Analg..

[50] Adami C, Lardone E, Monticelli P (2019). Inter-rater and inter-device reliability of mechanical thresholds measurement with the Electronic von Frey Anaesthesiometer and the SMALGO in healthy cats. J. Feline Med. Surg..

[51] Walton MB, Cowderoy E, Lascelles D, Innes JF (2013). Evaluation of construct and criterion validity for the 'Liverpool Osteoarthritis in Dogs' (LOAD) clinical metrology instrument and comparison to two other instruments. PLoS ONE.

[52] Hercock CA, Pinchbeck G, Giejda A, Clegg PD, Innes JF (2009). Validation of a client-based clinical metrology instrument for the evaluation of canine elbow osteoarthritis. J. Small Anim. Pract..

[53] Brown DC, Boston RC, Farrar JT (2013). Comparison of force plate gait analysis and owner assessment of pain using the Canine Brief Pain Inventory in dogs with osteoarthritis. J. Vet. Internal Med. Am. Coll. Vet. Internal Med..

[54] Brown DC, Boston RC, Coyne JC, Farrar JT (2008). Ability of the canine brief pain inventory to detect response to treatment in dogs with osteoarthritis. J. Am. Vet. Med. Assoc..

[55] Inc, A. *Canine ART ELISA Kit (ECA0039)*, https://abclonal.com/elisa-kits/CanineARTELISAKit/ECA0039 (2020).

