# Supplementary material for: Serum artemin is not correlated with sensitivity within dogs with naturally occurring osteoarthritis pain
Source: Sci Rep. 2021 Mar 23;11:6682. doi: 10.1038/s41598-021-85976-y (PMC7988108; doi:10.1038/s41598-021-85976-y)
Supplement: Supplementary file 1 — Supplementary Information [file 41598_2021_85976_MOESM1_ESM.docx]

**Title:** Serum artemin is not correlated with sensitivity within dogs with naturally occurring osteoarthritis pain

**Authors:** Ankita Gupta^1,2^, Ludovica Chiavaccini^3^, Laura M. Minnema^1,2^, King Wa Chiu^1,2^, David Knazovicky^1,2^, Jonathan A. Hash^1,2^, Santosh K. Mishra^3,4^, and B. Duncan X. Lascelles^1,2,4,5,6, *^

*Correspondence to dxlascel@ncsu.edu

**Affiliations:** ^1^ Translational Research in Pain Program, North Carolina State University, Raleigh, NC 27606, United States. ^2^Department of Clinical Sciences, College of Veterinary Medicine, North Carolina State University, Raleigh, NC 27606, United States**.** ^3^Department of Molecular Biomedical Sciences, College of Veterinary Medicine, North Carolina State University, Raleigh, NC 27606, United States**.** ^4^Comparative Pain Research and Education Centre, North Carolina State University, Raleigh, NC 27606, United States. ^5^Thurston Arthritis Center, University of North Carolina at Chapel Hill, Chapel Hill, NC 27599, United States**.** ^6^Center for Translational Pain Medicine, Duke University, Durham, NC 27710, United States.

**Supplementary Table S1:** Additional demographic information: breeds of all osteoarthritis subjects (n, %).

| Variable | Sample size (n, %) |
| --- | --- |
| Breed | Alaskan Malamute (n=1, 2.32%)  American Staffordshire Terrier (n=2, 4.65%)  Australian Cattle Dog (n=1, 2.32%)  Australian Shepherd (n=3, 6.98%)  Border Collie (n=3, 6.98%)  Boxer (n=1, 2.32%)  Brittany Spaniel (n=1, 2.32%)  Catahoula Leopard (n=3, 6.98%)  English Pointer (n=1, 2.32%)  German Shepherd Dog (n=4, 9.30%)  Golden Retriever (n=2, 4.65%)  Great Dane (n=1, 2.32%)  Great Pyrenees (n=1, 2.32%)  Hound (n=1, 2.32%)  Labrador Retriever (n=4, 9.30%)  Mixed Breed Dog (n=11, 25.58%)  Shetland Sheepdog (n=1, 2.32%)  Siberian Husky (n=1, 2.32%)  Standard Poodle (n=1, 2.32%) |

**Supplementary Table S2:** Canine orthopedic exam template: scoring system for joint pain and muscle atrophy scores.

| Forelimb | ROM | | Pain | | Crepitus | | Effusion | | Thickening | |
| --- | --- | --- | --- | --- | --- | --- | --- | --- | --- | --- |
|  | L | R | L | R | L | R | L | R | L | R |
| Manus |  |  |  |  |  |  |  |  |  |  |
| Carpus |  |  |  |  |  |  |  |  |  |  |
| Elbow |  |  |  |  |  |  |  |  |  |  |
| Shoulder |  |  |  |  |  |  |  |  |  |  |

**Joint Evaluation:**

| Hindlimb | ROM | | Pain | | Crepitus | | Effusion | | Thickening | |
| --- | --- | --- | --- | --- | --- | --- | --- | --- | --- | --- |
|  | L | R | L | R | L | R | L | R | L | R |
| Pes |  |  |  |  |  |  |  |  |  |  |
| Tarsus |  |  |  |  |  |  |  |  |  |  |
| Stifle |  |  |  |  |  |  |  |  |  |  |
| Hip |  |  |  |  |  |  |  |  |  |  |

Key:

Range of motion:

0: normal

1: mild-moderate decreased

2: severely decreased

Pain based on manipulation:

0: Does not notice manipulation

1: Orients to site on manipulation, does not resist or mild resistance (mild)

2: Orients to site, slight objection to manipulation (moderate)

3: Withdraws from manipulation, may vocalize, may turn to guard area (significant)

4: Tried to escape from manipulation, or prevent manipulation, may bite or show aggression on manipulation (severe)

Crepitus:

0: none, no crunching

1: mild, only feel crunching sometimes

2: moderate, crunching felt always, may be painful

3: severe, can feel and hear crunching, may be painful

Effusion:

0: none, no fluid pocket felt

1: mild, small fluid pocket felt only on palpation

2: moderate, prominent on palpation

3: severe, may see visible fluid pocket


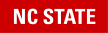


Copyright 2019 North Carolina State University, Translational Research in Pain (TRiP)

Thickening:

0: none, can feel all anatomic structures easily

1: mild, less defined anatomic structures

2: moderate, can slightly define anatomic structures

3: severe, can no longer feel anatomic structures

**Muscle Evaluation:**

| Muscles | R Forelimb | L Forelimb | Muscles | R Hindlimb | L Hindlimb |
| --- | --- | --- | --- | --- | --- |
| Supraspinatus |  |  | Gluteals |  |  |
| Infraspinatus |  |  | Semimembranous/  Semitendinous |  |  |
| Triceps Brachii |  |  | Biceps Femoris |  |  |
| Biceps Brachii |  |  | Quadriceps Femoris |  |  |
| Antebrachium |  |  | Crus |  |  |

Key:

Muscle atrophy:

0: None, normal, symmetric with opposite limb

1: mild muscle loss felt on palpation

2: moderate muscle loss is felt and slightly visible

3: severe, muscle loss is visible can palpate one or more underlying muscles


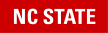


Copyright 2019 North Carolina State University, Translational Research in Pain (TRiP)

**Supplementary Figure S1:** Scatterplot of serum ARTN concentrations (ng/mL) versus body weight (kgs) in dogs with naturally occurring osteoarthritis-associated pain (r=-0.08, p=0.63). ARTN, artemin.

**
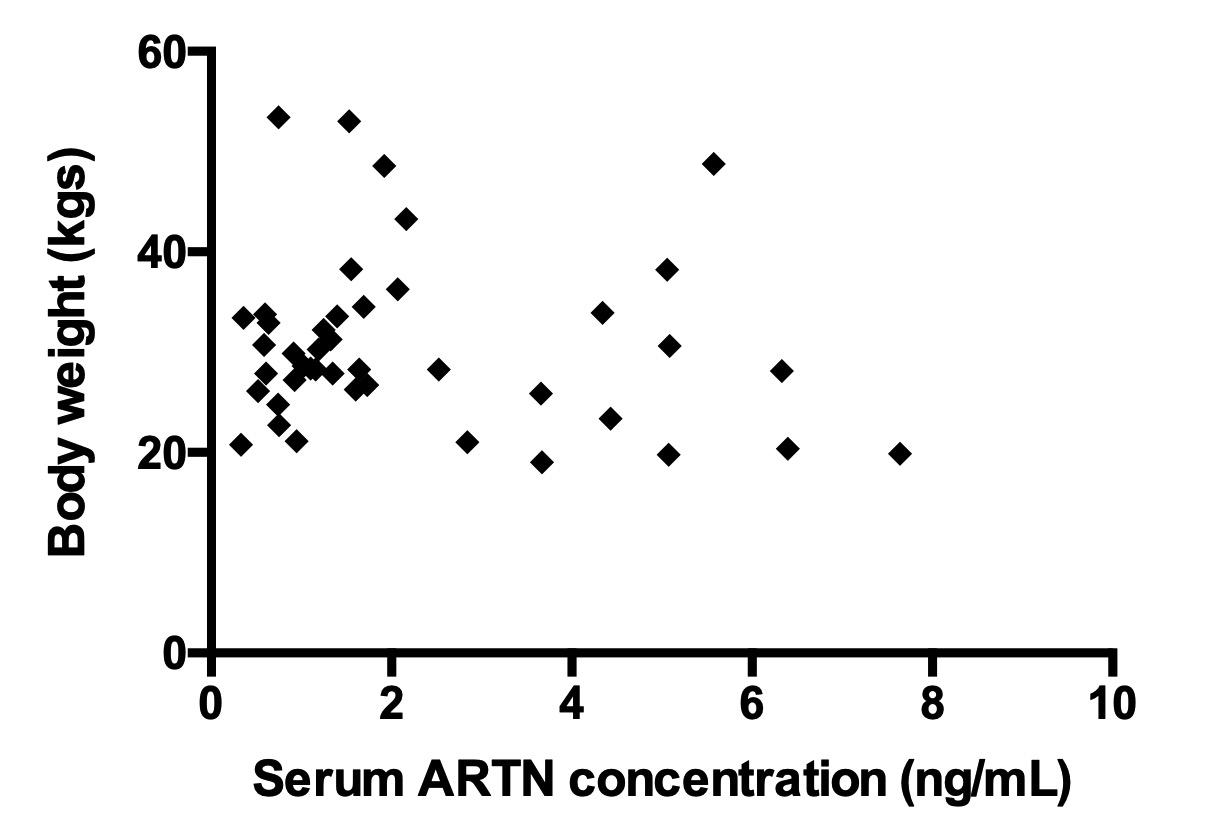
**

**Supplementary Figure S2:** Scatterplot of mechanical threshold (grams) and thermal latency (secs) in dogs with naturally occurring osteoarthritis-associated pain (r=51, p<0.001).


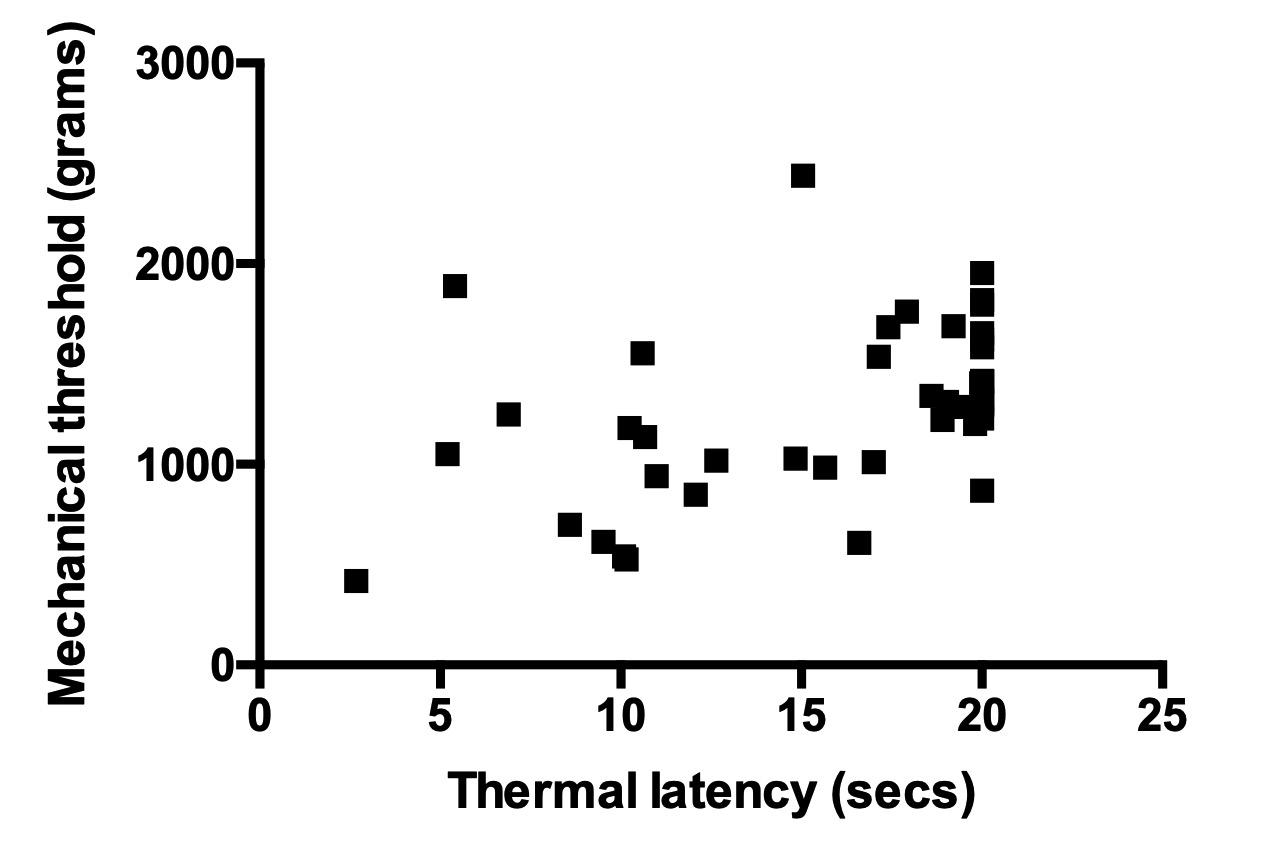


**Supplementary Figure S3:** Arrive and consort diagram to illustrate the study design.

Assessed for eligibility (n=74)

- Knazovicky D et al. 2016 study (n=31)
- Unpublished study (n=43)

Excluded (n=31)

- Not meeting inclusion criteria (n=25)
- Hemolyzed serum samples (n=6)
- Incomplete CMI data (LOAD n=2, CBPI n=1)

Screening (n=43)

- Serum collection for ARTN assessment
- Orthopedic exam findings
- CMI (LOAD n=41, CBPI n=42)
- Quantitative Sensory Testing

Analysed (n= 43)

- Multivariable regression analysis
- Spearman Correlations
